# Stroke progression index as a dynamic metric is associated with functional outcome in progressive ischemic stroke with large vessel occlusion

**DOI:** 10.3389/fneur.2026.1810149

**Published:** 2026-04-16

**Authors:** Bang Liu, Qishuo Yang, Ning Han, Kuochang Yin, Liang Ma, Wentao Yao, Qiang Sun, Yanzhao Xie, Guodong Xu

**Affiliations:** 1 Graduate School, Hebei North University, Zhangjiakou, China; 2 Department of Neurology, Hebei General Hospital Affiliated to Hebei Medical University, Shijiazhuang, China

**Keywords:** endovascular treatment, functional outcome, large vessel occlusion, progressive ischemic stroke, stroke progression index (SPI)

## Abstract

**Objective:**

Current evaluation of progressive ischemic stroke (PIS) with large vessel occlusion (LVO) relies on static clinical or imaging measures and lacks quantitative assessment of dynamic neurological deterioration. We proposed a novel metric, stroke progression index (SPI), to assess its association with clinical outcomes and benefit from endovascular treatment (EVT), and to explore its potential clinical relevance.

**Methods:**

We retrospectively enrolled consecutive LVO-PIS patients treated at our center between January 2022 and June 2025, limited to those with large-artery atherosclerosis. SPI was derived from National Institutes of Health Stroke Scale (NIHSS) change and progression time using a mathematically adjusted ratio. The primary endpoint was poor outcome at 90 days (mRS 3–6). We analyzed the association between SPI and outcomes, evaluated the effect of EVT, and explored relationships between SPI and disease course–related factors.

**Results:**

Among 190 patients, 44.2% (84/190) had a poor 90-day outcome, and 63.2% (120/190) received EVT. Regression analysis identified SPI as an independent risk factor for poor outcome (OR 1.223, 95% CI 1.100–1.360; *p* < 0.001). Adding SPI to a baseline model with traditional clinical variables improved discrimination (AUC 0.654 to 0.757; DeLong *p* < 0.001). In the EVT subgroup, SPI remained an independent risk factor (OR 1.134; 95% CI 1.018–1.263; *p* = 0.023). In treatment-effect analyses, lower SPI was associated with greater absolute benefit from EVT. Absolute risk reduction (ARR) was approximately 30% at SPI ≈11 and 10% at SPI ≈ 20. Exploratory analyses showed overall higher SPI in early than delayed progression, with poor outcomes being more common in patients with high SPI and poor collateral status.

**Conclusion:**

SPI is an independent prognostic risk factor in LVO-PIS that captures disease dynamics. The benefit of EVT varied across different SPI levels, suggesting that SPI may help inform clinical decision-making. These findings require further validation in larger, multicenter studies.

## Background

Progressive ischemic stroke (PIS) is defined as acute ischemic stroke with an increase of ≥4 points on the National Institutes of Health Stroke Scale (NIHSS) within 7 days of admission compared with baseline (1–4). PIS represents a clinical subtype of ischemic stroke, with a reported incidence of approximately 10–40%, and is strongly associated with mortality and poor functional outcomes at 3 months after stroke onset (5).

In clinical practice, a subset of patients with PIS have large vessel occlusion on admission (LVO-PIS). This subtype is commonly associated with chronic vascular lesions caused by large-artery atherosclerosis and often presents with mild initial symptoms or beyond the conventional time window for mechanical thrombectomy (6–8). As a result, these patients are frequently managed with medical therapy initially, with endovascular treatment (EVT) considered only after neurological deterioration occurs. Previous studies have suggested that EVT may be safe and beneficial in selected LVO-PIS populations under specific conditions (9–13). However, current assessments primarily rely on static indicators, such as infarct extent, occlusion site, NIHSS score after progression, and cerebral perfusion imaging (8, 14–17). The lack of effective metrics to quantify the dynamic progression process limits accurate prognostic evaluation and makes it difficult to determine optimal timing of intervention.

Therefore, we investigated patients with LVO-PIS treated at our center and proposed a novel metric, the stroke progression index (SPI), to assess its association with clinical outcomes and the benefit of EVT, and to explore its potential clinical value.

## Methods

### Study design and patient population

This was a single-center, retrospective cohort study that consecutively included patients with progressive ischemic stroke who were treated in the Department of Neurology at our hospital between January 2022 and June 2025.

Inclusion criteria were as follows: age ≥18 years; progressive neurological deterioration that occurred within 7 days after stroke onset despite standard medical therapy; and large vessel occlusion confirmed by computed tomography angiography (CTA), magnetic resonance angiography (MRA), or digital subtraction angiography (DSA). Eligible occlusion sites included the internal carotid artery (ICA), middle cerebral artery (MCA), vertebral artery (VA), or basilar artery (BA). To confirm that the occluded vessel was the culprit vessel, all cases were independently reviewed by two experienced neurointerventional specialists.

Exclusion criteria included large vessel occlusion caused by etiologies other than large-artery atherosclerosis; concomitant intracranial hemorrhage or other intracranial pathologies; severe systemic comorbidities that could substantially affect outcome assessment (such as advanced malignancy or severe infection); and incomplete clinical course documentation, National Institutes of Health Stroke Scale (NIHSS) records, imaging data, or follow-up information.

The study protocol was approved by the local institutional ethics committee (approval number: 2026-LW-013). Written informed consent was obtained from all patients or their legally authorized representatives.

### Clinical data collection

Clinical data were systematically collected, including demographic characteristics (age and sex), medical history (hypertension, diabetes mellitus, hyperlipidemia, atrial fibrillation, and prior ischemic stroke), and SPI-related variables, including baseline National Institutes of Health Stroke Scale score (NIHSS_baseline_), NIHSS score at progression (NIHSS_progression_), and time from symptom onset to neurological progression (Δ*t*). Treatment modalities (intravenous thrombolysis [IVT] and EVT), imaging data, and the modified Rankin Scale (mRS) score at 90 days were also recorded.

### Treatment strategies

All patients received standard medical therapy in accordance with the 2023 Chinese guidelines for the diagnosis and treatment of acute ischemic stroke, including antiplatelet therapy, statins, and basic management of blood pressure and blood glucose. Eligible patients received IVT according to guideline recommendations (18). The decision to perform EVT was made by physicians with more than 10 years of neurointerventional experience. EVT was performed according to the 2023 Chinese guidelines for endovascular treatment of acute ischemic stroke using standard thrombectomy techniques, including stent retriever thrombectomy or combined aspiration approaches (19). All procedures were conducted by an experienced neurointerventional team.

### Definitions

Progressive Ischemic Stroke with Large Vessel Occlusion (LVO-PIS) was defined as acute ischemic stroke with large vessel occlusion (including ICA/MCA/BA/VA) in which patients developed progressive neurological deterioration within 7 days after admission despite standard medical therapy, manifested as an increase of ≥4 points in the NIHSS score compared with baseline (ΔNIHSS ≥4) (2, 20). Imaging confirmed new infarct lesions relative to baseline imaging, and the occluded vessel was identified as the culprit vessel. Neurological deterioration attributable to other causes, such as re-occlusion or intracranial hemorrhage, was excluded.

Stroke progression index (SPI) was defined as a metric to quantify neurological progression, calculated from NIHSS-related parameters and progression time using a mathematically adjusted ratio. The detailed formula is provided below. The baseline NIHSS score (NIHSS_baseline_) was defined as the NIHSS score assessed at stroke onset, whereas the NIHSS score at progression (NIHSS_progression_) was defined as the NIHSS score assessed at the time of the first documented neurological deterioration (ΔNIHSS ≥4). The change in NIHSS score (ΔNIHSS) was defined as the magnitude of NIHSS change from stroke onset to the first occurrence of neurological deterioration, calculated as the difference between NIHSS_progression_ and NIHSS_baseline_. Progression time (Δ*t*) was defined as the time interval from stroke onset to this first documented neurological deterioration (ΔNIHSS ≥4), measured in hours.

Regarding the timing of progression, early progression was defined as neurological deterioration occurring within 24 h after stroke onset, whereas delayed progression was defined as deterioration occurring between 24 h and 7 days after onset (21). Functional outcome was assessed using the modified Rankin Scale (mRS) at 90-day follow-up, with poor outcome defined as an mRS score of 3–6 and good outcome defined as 0–2.

In patients treated with EVT, collateral circulation was assessed using DSA and graded according to the ASITN/SIR scale (range, 0–4), with higher grades indicating better collateral status. Successful reperfusion was defined as a modified Thrombolysis in Cerebral Infarction (mTICI) score of ≥2b.

NIHSS scores were obtained from routine clinical records and assessed by NIHSS-certified neurology staff in accordance with standard stroke care practice at admission, during routine inpatient evaluations, and at the time of neurological deterioration. NIHSS_baseline_ was assessed at the initial clinical evaluation by a neurology resident certified in NIHSS scoring and confirmed by an attending neurointerventional physician. When neurological deterioration was first observed by the resident, attending physician, or other on-call staff, the event was documented in the medical record, and the NIHSS score was updated and recorded as NIHSS_progression_. NIHSS at progression was assessed by NIHSS-certified personnel according to standard procedures and confirmed by the attending physician when applicable. If there was disagreement during the NIHSS assessment, another attending physician performed an additional assessment to ensure consistency.

### Calculation of the stroke progression index

The SPI proposed in this study was calculated using the following formula, designed to quantify the temporal dimension of neurological deterioration.


$$\mathrm{SPI}=\frac{\Delta \text{NIHSS}}{\Delta t^{0.5}}\ln(1+\frac{{\text{NIHSS}}_{\text{progression}}}{{\text{NIHSS}}_{\text{baseline}}}).$$

To mitigate the undue influence of extremely long or short time intervals on the metric, Δ*t* was transformed using a 0.5 power (square-root transformation). To differentiate the clinical significance of identical absolute neurological progression across patients with different baseline severities, a logarithmic relative burden term was incorporated. The complete derivation and theoretical rationale are provided in the Supplementary material.

### Statistical analysis

All statistical analyses were performed using SPSS version 30.0 (IBM Corp., Armonk, NY) and R software version 4.5.1. Continuous variables with a normal distribution are presented as mean ± standard deviation and were compared using the independent-samples *t* test. Non-normally distributed continuous variables are presented as median (interquartile range) and were compared using the Mann–Whitney *U* test. Categorical variables are expressed as counts (percentages) and were compared using the *χ*^2^ test or Fisher’s exact test, as appropriate.

Univariable analyses were first conducted to identify potential factors associated with 90-day functional outcomes, with variables showing *p* < 0.10 entered into multivariable analyses. Multivariable binary logistic regression models were then constructed to evaluate the independent association between SPI and 90-day functional outcome. Variance inflation factor (VIF) diagnostics were performed for SPI and its component variables, and only SPI was included in the final models to avoid simultaneous inclusion of highly collinear components and thereby reduce structural multicollinearity.

### Subgroup analysis

Among patients who received EVT, interaction models were further constructed to evaluate whether the treatment benefit of EVT varied according to SPI. Based on these models, absolute risk reduction (ARR) and the corresponding number needed to treat (NNT) were estimated across different SPI levels, and curves showing EVT benefit across the SPI spectrum were generated to identify potential thresholds for stratification.

SPI distributions, ASITN/SIR collateral grades, and outcome-related characteristics were further compared between patients with good and poor outcomes. Spearman rank correlation analysis was performed to assess the relationship between SPI and collateral status. In addition, SPI was further analyzed according to the timing of progression (early versus delayed) to explore the pathophysiological characteristics of infarct progression.

## Results

### Overall patient characteristics

A total of 190 patients with LVO-PIS treated in the Department of Neurology at Hebei General Hospital between January 2022 and June 2025 were included. At 90 days, 84 patients (44.21%) had a poor functional outcome (mRS 3–6). EVT was performed in 63.16% of patients (120/190).

Baseline characteristics are summarized in Table 1. Compared with patients with good outcomes (*n* = 106), those with poor outcomes (*n* = 84) were older (64.58 ± 12.83 vs. 59.84 ± 12.40 years; *p* = 0.011), had a higher prevalence of prior ischemic stroke (31.0% vs. 17.0%; *p* = 0.036), and more frequently had anterior circulation occlusion (84.5% vs. 67.0%; *p* = 0.009). The proportion of patients receiving EVT was significantly lower in the poor-outcome group (50.0% vs. 73.6%; *p* = 0.001).

**Table 1:** Baseline characteristics.

Variable	Good outcome (*n* = 106)	Poor outcome (*n* = 84)	*p* value
Age, years	59.84 ± 12.40	64.58 ± 12.83	0.011
Male sex	80 (75.5)	54 (64.3)	0.129
Hypertension	72 (67.9)	59 (70.2)	0.854
Diabetes mellitus	23 (21.7)	27 (32.1)	0.145
Hyperlipidemia	17 (16.0)	9 (10.7)	0.397
Atrial fibrillation	8 (7.5)	10 (11.9)	0.442
Prior ischemic stroke	18 (17.0)	26 (31.0)	0.036
Smoking	55 (51.9)	34 (40.5)	0.156
Anterior circulation occlusion	71 (67.0)	71 (84.5)	0.009
Early progression	53 (50.0)	51 (60.7)	0.185
EVT	78 (73.6)	42 (50.0)	0.001
IVT	30 (28.3)	33 (39.3)	0.149
NIHSS_baseline_	2.50 (2.00–4.00)	3.00 (2.00–5.00)	0.066
NIHSS_progression_	9.50 (7.00–12.00)	13.00 (10.75–15.00)	<0.001
ΔNIHSS	6.00 (4.00–9.00)	9.00 (7.00–12.00)	<0.001
Δ*t*, hours	26.50 (6.00–76.25)	14.00 (5.00–45.00)	0.045
SPI	2.05 (1.04–3.65)	3.89 (1.62–8.38)	<0.001

Values are presented as mean ± SD, median (interquartile range), or *n* (%). Abbreviations: EVT, endovascular treatment; IVT, intravenous thrombolysis. NIHSS, National Institutes of Health Stroke Scale; NIHSS_baseline_, NIHSS score at stroke onset; NIHSS_progression_, NIHSS score at first neurological deterioration; ΔNIHSS, change in NIHSS score from onset to progression; Δ*t*, time from onset to progression; SPI, stroke progression index.

Regarding disease progression, patients with poor outcomes had a shorter Δ*t* (14.00 [5.00–45.00] h vs. 26.50 [6.00–76.25] h; *p* = 0.045) and a higher SPI (3.89 [1.62–8.38] vs. 2.05 [1.04–3.65]; *p* < 0.001). Both the NIHSS_progression_ and ΔNIHSS were significantly higher in the poor-outcome group (both *p* < 0.001).

### Univariable analysis and collinearity diagnostics

Univariable logistic regression analyses (Supplementary Table 1) showed that age (OR 1.031; 95% CI 1.007–1.056; *p* = 0.012), anterior circulation occlusion (OR 2.692; 95% CI 1.315–5.512; *p* = 0.007), NIHSS_baseline_ (OR 1.177; 95% CI 1.020–1.357; *p* = 0.025), NIHSS_progression_ (OR 1.189; 95% CI 1.100–1.286; *p* < 0.001), ΔNIHSS (OR 1.155; 95% CI 1.069–1.248; *p* < 0.001), and SPI (OR 1.155; 95% CI 1.069–1.248; *p* < 0.001) were significantly associated with poor outcome. EVT was inversely associated with poor outcome (OR 0.359; 95% CI 0.195–0.659; *p* < 0.001). Δ*t* was also inversely associated with poor outcome (OR 0.993; 95% CI 0.987–1.000; *p* = 0.041).

Collinearity diagnostics (Supplementary Table 2) indicated substantial multicollinearity among NIHSS_baseline_, NIHSS_progression_, and ΔNIHSS (variance inflation factor [VIF] > 10), whereas no substantial collinearity was observed for SPI (VIF 3.194) or Δ*t* (VIF 1.553). Accordingly, highly collinear variables were not included simultaneously in subsequent multivariable models.

### Interaction analysis between SPI and anterior circulation

After anterior circulation occlusion was included as a covariate in the multivariable model, age (*p* = 0.025), SPI (OR 1.269; 95% CI 1.133–1.421; *p* < 0.001), anterior circulation occlusion (OR 3.589; 95% CI 1.491–8.640; *p* = 0.004), and EVT (OR 0.300; 95% CI 0.143–0.629; *p* = 0.001) were independently associated with poor outcome, whereas Δ*t* did not reach statistical significance (Supplementary Table 3).

Given the known differences in clinical characteristics and NIHSS score sensitivity between anterior and posterior circulation strokes, an interaction-term logistic regression model was further constructed to assess whether the prognostic effect of SPI differed by circulation territory (Supplementary Table 4). SPI remained significantly associated with poor outcome (OR 1.214; 95% CI 1.063–1.387; *p* = 0.004), as did anterior circulation occlusion (OR 4.917; 95% CI 1.457–16.601; *p* = 0.010), whereas the interaction term was not statistically significant (OR 0.960; 95% CI 0.810–1.137; *p* = 0.635), suggesting that the effect of SPI was consistent across anterior and posterior circulation.

Comparison of the model including anterior circulation occlusion (Supplementary Table 3) with the corresponding model without this variable (Table 2) showed that the estimated OR for SPI changed by less than 5% (approximately 3.8%), with no change in effect direction or statistical significance. Accordingly, anterior circulation occlusion was not retained in subsequent multivariable analyses.

**Table 2:** Multivariable logistic regression analysis.

Variable	*B*	Standard error	Wald *χ*^2^	OR	95% CI	*p* value
Age	0.029	0.014	4.371	1.030	1.002–1.059	0.037
Male sex	−0.279	0.376	0.553	0.756	0.362–1.579	0.457
Prior ischemic stroke	0.433	0.395	1.201	1.542	0.711–3.348	0.273
EVT	−1.336	0.363	13.552	0.263	0.129–0.535	<0.001
Δ*t*	−0.001	0.004	0.120	0.999	0.991–1.007	0.729
SPI	0.201	0.054	13.812	1.223	1.100–1.360	<0.001

EVT, endovascular treatment; Δ*t*, time from stroke onset to progression; SPI, stroke progression index.

### Multivariable analysis

Based on the interaction analysis, anterior circulation occlusion was not retained in the final model. A final multivariable logistic regression model was therefore constructed (Table 2). SPI was independently associated with poor outcome (OR 1.223, 95% CI 1.100–1.360; *p* < 0.001). Age was identified as a risk factor (OR 1.030, 95% CI 1.002–1.059; *p* = 0.037), whereas EVT was a protective factor (OR 0.263, 95% CI 0.129–0.535; *p* < 0.001). Δ*t*, sex, and prior ischemic stroke were not statistically significant in this model.

### Predictive performance of SPI

**Figure 1:**
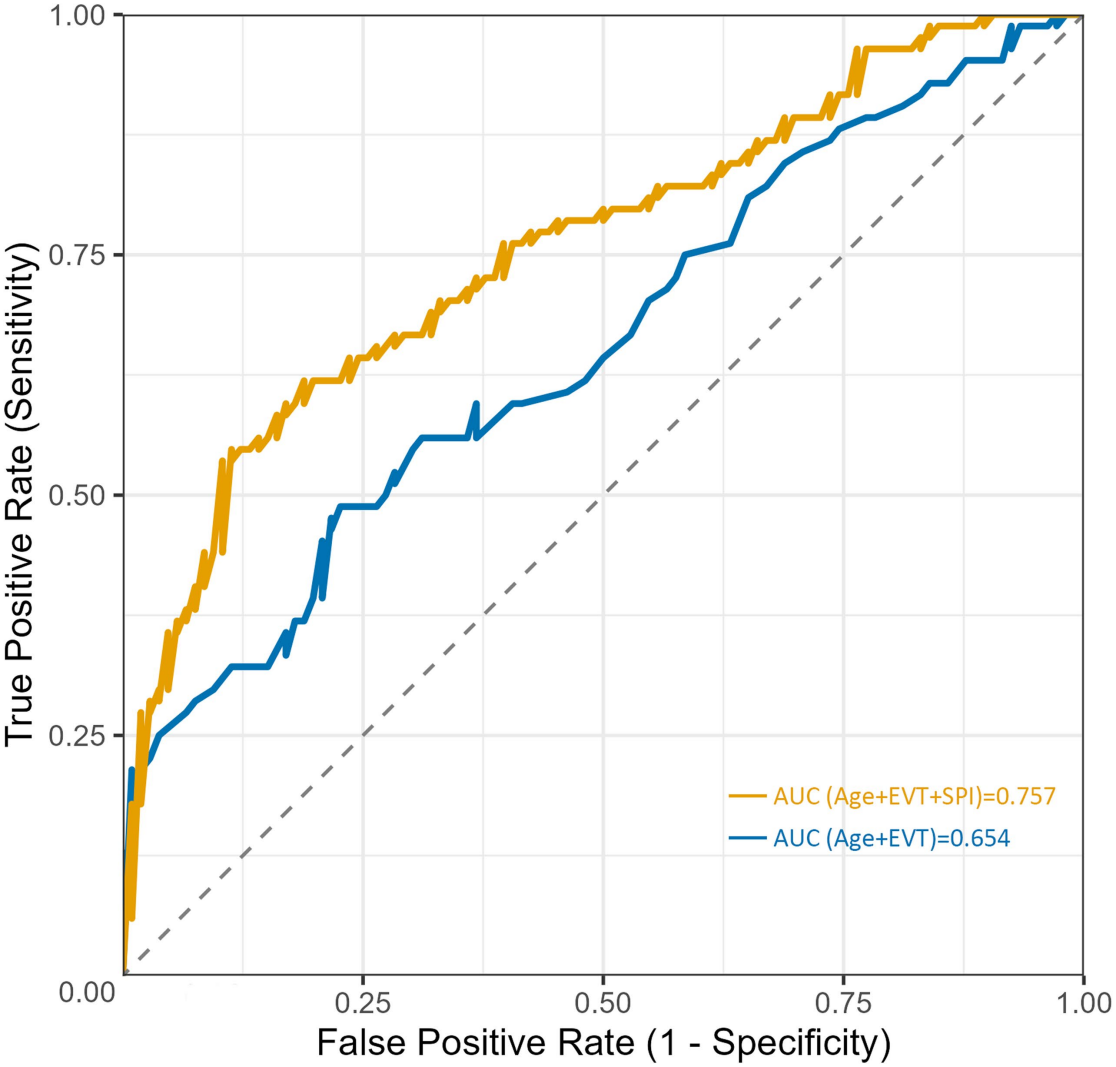
Receiver operating characteristic (ROC) curves comparing predictive models with and without SPI. The model including age, EVT, and SPI (orange line) showed better discriminative performance than the model including age and EVT alone (blue line). The corresponding areas under the curve (AUCs) were 0.757 and 0.654, respectively.

ROC curve analyses are shown in Figure 1. Using poor outcome as the dependent variable, the model without SPI (age + EVT) yielded an AUC of 0.654 (95% CI 0.574–0.729). After SPI was added (age + EVT + SPI), the AUC increased to 0.757 (95% CI 0.684–0.822). The absolute difference in AUC between the two models was 0.103, and the incremental improvement was statistically significant according to the DeLong test (*χ*^2^(1) = 26.915, *p* < 0.001), indicating that SPI provided additional discriminative ability (Supplementary Table 5).

### EVT subgroup analysis

#### Clinical characteristics and multivariable analysis

Among EVT-treated patients (*n* = 120), those with poor outcome had significantly higher NIHSS scores at progression and greater ΔNIHSS (both *p* < 0.001), higher SPI (4.86 [2.04–9.71] vs. 2.76 [1.46–4.13], *p* = 0.001), and lower collateral grades (*p* < 0.001) compared with patients with good outcome (Supplementary Table 6).

In univariable analyses, prior ischemic stroke (OR 2.720; 95% CI 1.023–7.231; *p* = 0.045), NIHSS_progression_ (OR 1.127; 95% CI 1.036–1.227; *p* = 0.006), ΔNIHSS (OR 1.160; 95% CI 1.058–1.272; *p* = 0.002), SPI (OR 1.180; 95% CI 1.072–1.298; *p* = 0.001), and ASITN/SIR collateral grade (OR 0.252;95% CI 0.134–0.473; *p* < 0.001) were associated with outcome (Supplementary Table 7).

After variables with high collinearity (VIF > 5) were excluded (Supplementary Table 8), the multivariable model included prior ischemic stroke, SPI, and ASITN/SIR collateral grade. All three remained independently associated with outcome: prior ischemic stroke (OR 3.435, 95% CI 1.055–11.181, *p* = 0.040) and SPI (OR 1.134, 95% CI 1.018–1.263, *p* = 0.023) were identified as risk factors, whereas ASITN/SIR collateral grade was a protective factor (OR 0.269, 95% CI 0.137–0.531, *p* < 0.001) (Table 3).

**Table 3:** Multivariable logistic regression analysis in EVT-treated patients.

Variable	*B*	Standard error	Wald *χ*^2^	OR	95% CI	*p* value
Prior ischemic stroke	1.234	0.602	4.201	3.435	1.055–11.181	0.040
SPI	0.125	0.055	5.193	1.134	1.018–1.263	0.023
ASITN/SIR collateral grade	−1.312	0.346	14.371	0.269	0.137–0.531	<0.001

SPI, stroke progression index.

#### Exploratory stratification of EVT benefit based on SPI

**Figure 2:**
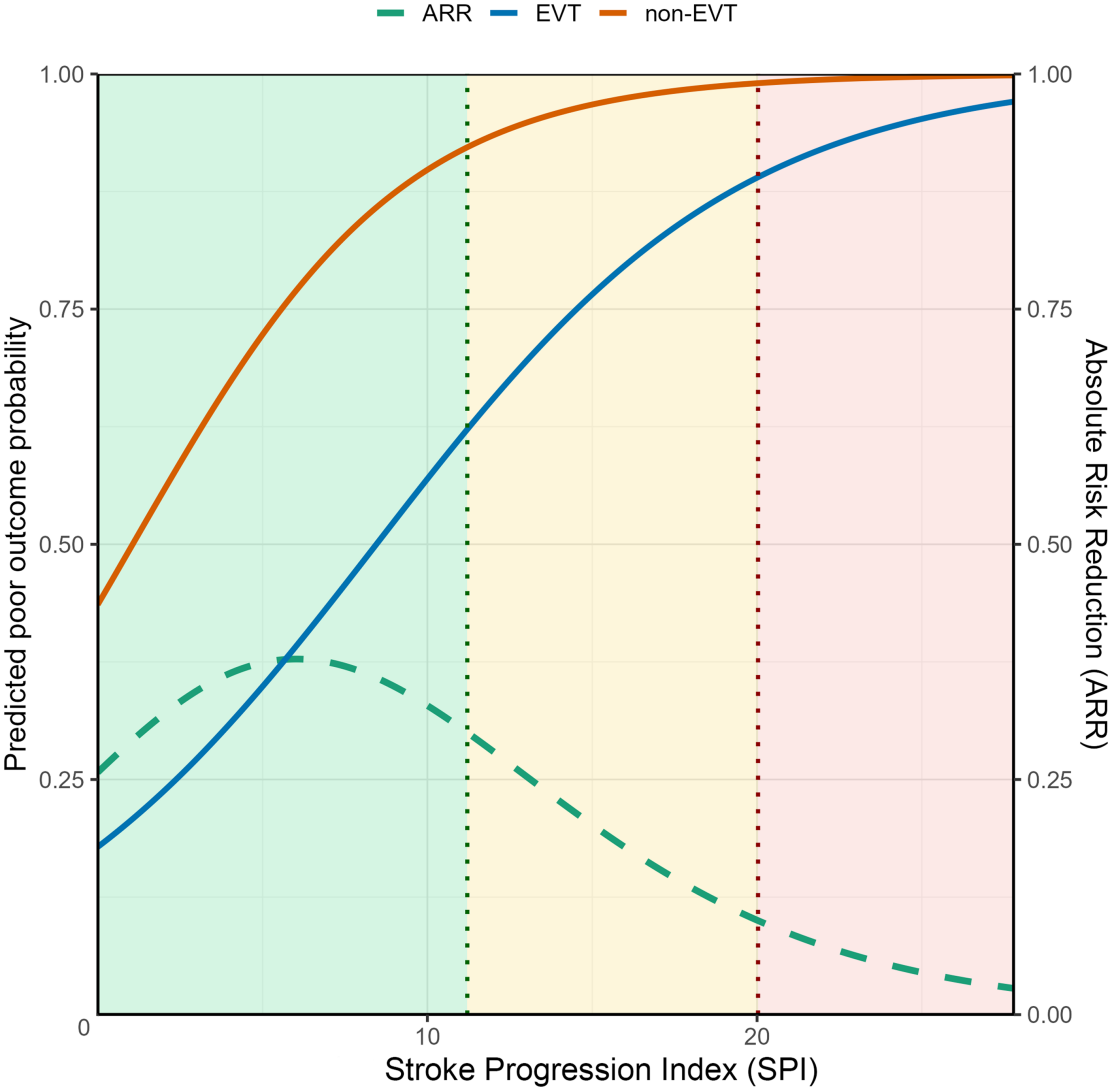
Predicted risk of poor outcome under EVT and non-EVT management across SPI values. Predicted probabilities of poor outcome were estimated from a logistic regression model including SPI, EVT, and their interaction. The dashed curve represents absolute risk reduction (ARR). Shaded regions indicate ARR-based strata: green, ARR > 30%; yellow, ARR 10–30%; red, ARR < 10%. Vertical dotted lines denote SPI values of approximately 11 and 20.

Based on a logistic regression model incorporating SPI, EVT, and their interaction term, risk curves were generated for patients treated with EVT and those managed without EVT, and the ARR and NNT were estimated (Figure 2). Overall, ARR showed a decreasing trend with increasing SPI values.

Using ARR-based thresholds, two inflection points were identified at approximately SPI ≈ 11 (ARR ≈ 30%) and SPI ≈ 20 (ARR ≈ 10%), which allowed construction of a three-tier stratification framework (Table 4). In general, lower SPI values were associated with greater absolute benefit from EVT, whereas the net clinical benefit of EVT appeared to diminish substantially at higher SPI levels, particularly beyond approximately 20.

**Table 4:** Stratification of ARR and NNT across SPI levels.

SPI range	ARR	NNT	Interpretation of clinical benefit
SPI < 11	>30%	≤3–4	High-benefit stratum: EVT was associated with the largest absolute risk reduction in this range in the observational cohort.
11 ≤ SPI ≤ 20	10–30%	≈4–10	Intermediate-benefit stratum: EVT remained associated with benefit, which gradually decreased with increasing SPI values.
SPI > 20	<10%	≥10	Low-benefit stratum: The absolute benefit associated with EVT appeared attenuated at higher SPI levels.

SPI, stroke progression index; ARR, absolute risk reduction; NNT, number needed to treat; EVT, endovascular treatment. This stratification is exploratory and intended for descriptive interpretation rather than direct clinical decision-making.

#### Exploration of the associations between SPI, collateral status, and timing of progression

**Figure 3:**
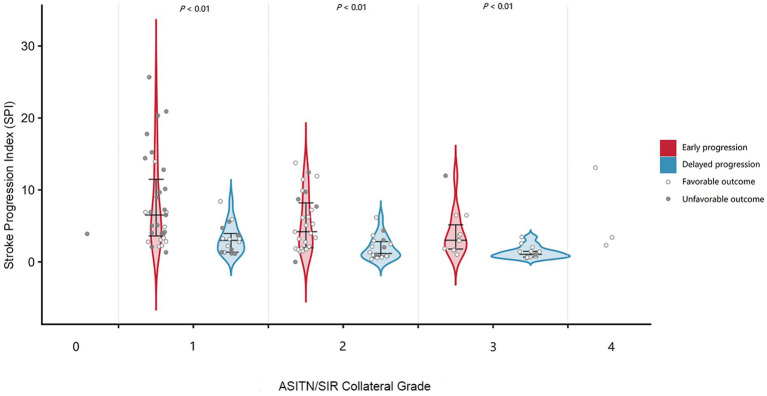
Distribution of SPI across ASITN/SIR collateral grades stratified by timing of progression. Violin plots show the distribution of SPI according to ASITN/SIR collateral grade and timing of progression (early vs. delayed). Individual data points are shown. Colors indicate timing of progression (red, early; blue, delayed), and symbols indicate functional outcome (filled circles, unfavorable; open circles, favorable).

Spearman’s rank correlation analysis demonstrated a moderate negative correlation between SPI and ASITN/SIR collateral grading (*ρ* = −0.336, *p* < 0.001). To further explore the relationships among collateral status, SPI, and functional outcome, patients were stratified according to ASITN/SIR collateral grade and timing of progression (early vs. delayed), and the distribution of SPI was visualized (Figure 3). Patients in the ASITN/SIR grades 0 and 4 were not included in the stratified analysis because of limited sample sizes.

Within ASITN/SIR grades 1–3, SPI values were significantly higher in the early progression group than in the delayed progression group at each collateral grade (all *p* < 0.01). In addition, SPI decreased as collateral grade increased. In the scatter plot, patients with poor outcomes were more commonly located in regions with higher SPI values, lower collateral grades, and early progression (Figure 3). Group sizes for each ASITN/SIR grade and progression timing are shown in Supplementary Table 9.

## Discussion

In this single-center cohort study of LVO-PIS, we introduced a dynamic metric—stroke progression index (SPI)—to capture the temporal dimension of neurological deterioration and systematically evaluated its clinical relevance. Incorporating into a baseline model including conventional clinical factors (age and EVT) significantly improved discrimination for unfavorable 90-day outcomes, with the AUC increasing from 0.654 to 0.757 (DeLong test, *p* < 0.001); SPI remained independently associated with outcome in the EVT-treated subgroup. The prognostic association of SPI appeared consistent across vascular territories, as interaction testing between SPI and anterior versus posterior circulation was not statistically significant, suggesting similar prognostic implications across different vascular distributions. In addition, SPI was closely associated with collateral status assessed by the ASITN/SIR grading system and with the timing of progression. Taken together, these findings indicate that incorporating SPI into the assessment of LVO-PIS may improve characterization of disease progression and provide complementary information for prognostic evaluation.

Beyond age and EVT, SPI improved overall discrimination and remained independently associated with 90-day outcomes. Although this study was not designed as a formal model-comparison analysis, the absolute AUC values and their comparison using the DeLong test help show that the incremental value of SPI is not only statistically significant but also potentially clinically meaningful. In future extensions of this work, additional analyses such as model calibration may be performed to further explore potential nonlinear relationships.

From a conceptual perspective, the SPI should reflect the time sensitivity of treatment benefit. Patients with slower progression (low SPI) may preserve salvageable brain tissue for a longer period and therefore retain potential benefit from EVT even in the presence of procedural delays, whereas in patients with rapid progression (high SPI), treatment benefit may diminish quickly if early recanalization cannot be achieved. Similar to previous studies, we further explored the practical significance of SPI in LVO-PIS across different clinical parameters (22, 23). In our study, the effect of EVT varied in a clinically meaningful manner across the spectrum of SPI, which was illustrated using continuous effect curves and summarized through risk-stratified estimates of absolute risk reduction and number needed to treat (ARR/NNT; Figure 2; Table 4). Given the observational nature of this cohort, these findings are interpreted as reflecting heterogeneity rather than causal effects under randomized conditions. Although SPI is not intended to replace imaging-based assessment, the progression it captures helps characterize patients’ time sensitivity and provides complementary information for clinical decision-making, particularly in situations where conventional imaging and clinical indicators do not support or rule out intervention and a balance between treatment timing must be weighed against expected benefit.

Previous studies have suggested that LVO-PIS is predominantly associated with large-artery atherosclerosis (LAA), whereas progression due to cardioembolism (CE) is relatively uncommon (6–8). This may be because collateral networks in CE are often sparse and cause severe deficits early (24); when neurological worsening occurs, it is more frequently attributable to early recurrent ischemic stroke (ERIS) rather than continued progression of the index lesion, making it difficult to classify as true PIS (25, 26). To reduce etiologic heterogeneity and focus on pathophysiology most consistent with PIS, we therefore restricted our cohort to patients with LAA.

In the setting of LAA, chronic hypoperfusion with varying degrees of collateral compensation constitutes a fragile equilibrium. *In situ* thrombosis, systemic hemodynamic compromise, inflammatory and endothelial responses, metabolic disturbances, and persistent thrombus burden may precipitate collateral failure, thereby driving conversion of penumbral tissue to infarct core (24, 27–31). Prior evidence has also linked poor collateral status to more rapid infarct expansion (32–34). Accordingly, we prespecified collateral status as a potential pathophysiologic determinant of SPI. Consistent with this hypothesis, SPI showed a moderate negative correlation with ASITN/SIR collateral grade (*ρ* = −0.336, *p* < 0.001), supporting the physiological notion that better collaterals are associated with slower progression. However, the moderate strength of this association also suggests that SPI is not merely a surrogate for collateral status.

We further stratified patients by timing of progression into early (within 24 h) and delayed (24 h to 7 days) progression groups (21). Based on the hypothesis that more stable collateral supply is associated with slower progression, we visualized SPI distributions stratified by collateral grade (Figure 3). Within each collateral stratum, SPI values were significantly higher in the early progression group than in the delayed progression group (all *p* < 0.01). Moreover, scatter plots showed that patients with unfavorable outcomes tended to cluster in regions characterized with higher SPI, lower collateral grade, and early progression. Combined with previous studies, microcirculatory adequacy, metabolic state, inflammatory milieu, and tissue tolerance may jointly influence the evolution of progressive ischemic stroke (35–38). Therefore, our findings suggest that SPI may represent a macroscopic phenotype integrating multiple factors and may thereby exhibit independent prognostic value.

For generalizability, we included patients with both anterior and posterior circulation occlusions. However, similar to previous PIS studies, posterior circulation cases were relatively uncommon, accounting for 25.26% (48/190) of the cohort, which limited the statistical power of territory-specific analyses (12, 13, 39). We therefore explicitly tested the interaction between vascular territory and SPI, and no significant effect modification was observed, suggesting that, within the current sample size, the direction of the association between SPI and outcome was generally consistent across vascular territories. These findings provide preliminary support for SPI as a broadly applicable risk metric across occlusion locations and treatment pathways. Nevertheless, given the limited number of posterior circulation cases, this result should be interpreted as a signal of robustness rather than definitive evidence, and the performance of SPI across different vascular territories warrants further evaluation in larger, multicenter cohorts with more balanced representation.

At the same time, there is currently no unified definition of progressive ischemic stroke (PIS) (4). Given that this study focused on neurological deterioration with clear clinical relevance, we adopted the most widely used criterion and one with a stronger association with outcome—an increase of ≥4 points in the NIHSS score from baseline—as the threshold for defining PIS. With respect to the time window for progression, previous studies have used observation periods ranging from 24 h to 7 days after onset, which limits comparability across studies (2, 15, 20). Based on prior literature and our institutional experience, we observed that a substantial proportion of clinically meaningful deterioration occurred between 24 h and 7 days, whereas events beyond 7 days were relatively uncommon and often difficult to attribute to the index atherosclerotic occlusion (e.g., new vascular events). Accordingly, defining PIS as an NIHSS increase of ≥4 points within a 7-day window allowed us to capture progression with practical relevance for clinical decision-making. This approach also aligns with real-world practice, as escalation of therapy in LVO-PIS is often determined over several days rather than within the first 24 h alone.

Previous studies have extensively investigated predictors of progressive ischemic stroke. Josef et al. reviewed quantitative baseline imaging markers, including early CT hypodensity, DWI/PWI lesion volumes and mismatch, as well as more stringent DWI volume and perfusion (CBF) thresholds within the malignant mismatch (MMI) framework (15). Other studies have identified anatomical factors—such as infarct location, occluded vessel site, and lesion size—as independent predictors (8, 16, 17). In addition, NIHSS scores assessed after progression have also been reported as independent risk factors for unfavorable outcome (9). However, these indicators essentially reflect disease status at a single time point and do not capture the dynamic course of disease progression. Moreover, although both NIHSS_baseline_ and ΔNIHSS showed strong statistical associations with prognosis in the univariable analysis of this study, consistent with previous similar studies, potential collinearity among variables still needed to be considered in further modeling to avoid interference with the evaluation of the independent effect of SPI (40–42).

Previous work examining NIHSS in relation to time has followed several complementary directions. Static NIHSS measurements (e.g., NIHSS at 24 h) have demonstrated robust predictive value for 90-day outcomes and often outperform absolute ΔNIHSS or baseline NIHSS, whereas proportional changes (ΔNIHSS × 100%/baseline NIHSS) have not consistently shown additional predictive benefit (43, 44). Although these studies were not conducted specifically in PIS populations, they offer important insight into the relationship between NIHSS-related parameters and clinical outcomes. Other studies have conceptualized neurological deterioration (ND) as a time-to-event process (i.e., time to ND), showing that irreversible ND tends to occur earlier than reversible ND, and that older age and higher baseline NIHSS are associated with shorter time to ND (45). This line of work highlights the importance of explicitly incorporating the temporal dimension into the assessment of ND. Building on this perspective, Meurer et al. reported a reanalysis of data from the NINDS tPA randomized trial and proposed the slope of NIHSS change within an early time window—that is, the rate of change in NIHSS per unit time—as a quantitative marker of disease progression (46). They demonstrated that the NIHSS decline rate within the first 2 h was strongly associated with 90-day functional outcome, emphasizing progression as a kinetic dimension of stroke evolution and providing a methodological precedent for the SPI proposed in the present study. Related concepts have also been explored in imaging-based analyses, where infarct formation has been quantified as infarct core volume divided by the time from onset to imaging. Although this approach focused on infarct evolution at stroke onset rather than PIS, its methodological rationale remains informative (47). In acute triage settings, Seners et al. proposed the ENDᵢ score, which uses occlusion site and thrombus length to estimate the risk of early neurological deterioration (END) in patients with mild LVO treated with intravenous thrombolysis alone (48).

Taken together, the SPI framework aligns with prior literature in treating time or speed not merely as a background descriptor but as an explicit analytical variable, while extending existing work in three respects: by focusing specifically on in-hospital PIS rather than broader acute stroke populations; by linking SPI to EVT benefit, thereby addressing the questions of whether and when to intervene; and by allowing complementary use with existing tools—such as ENDᵢ in the ultra-acute phase—to identify high-risk mild LVO patients and support consideration of bridging thrombectomy.

Our SPI can be regarded as an extension of the NIHSS into the temporal domain. Because it is fundamentally derived from clinical symptom assessment, it retains broad applicability across clinical settings. The metric does not rely on imaging data or specific hardware requirements and can therefore be used across institutions at different levels of care. Moreover, as a symptom-based quantitative measure, SPI can be readily calculated at the bedside once neurological deterioration is recognized, supporting its practical feasibility. With further validation in larger cohorts, its clinical utility may be enhanced by the development of simplified calculation tools to facilitate real-world implementation.

## Limitations

This study has several limitations. First, it was a single-center, retrospective analysis with a relatively limited sample size, and no formal *a priori* sample size calculation was performed. Therefore, the statistical power could not be directly assessed, and negative findings should be interpreted with caution, and the generalizability of the findings may be limited. Second, the calculation of SPI in this study depended on time points and NIHSS assessments documented in prior clinical records. Because these data were derived from routine clinical practice rather than collected according to a prospectively predefined assessment schedule, the frequency of repeated assessments was not fully standardized, and formal interrater reliability testing was not performed. Therefore, the SPI may have been affected by documentation bias and interrater variability. Third, imaging parameters (e.g., perfusion-defined penumbra or infarct core volume) were not incorporated for combined validation. In addition, external validation and prospective testing were not performed. Therefore, the reproducibility and clinical applicability of SPI require further confirmation in larger, multicenter, prospective studies.

## Conclusion

SPI, as a quantitative metric of disease progression is independently associated with prognosis in patients with LVO-PIS and captures the dynamic course of neurological deterioration. The benefit of EVT varied across different SPI levels, suggesting that SPI may provide supplementary information for clinical decision-making. SPI was also associated with collateral status and timing of progression. These findings warrant further validation in larger, multicenter cohorts.

## Data Availability

The raw data supporting the conclusions of this article will be made available by the authors, without undue reservation.
