# Supplementary material for: Stroke progression index as a dynamic metric is associated with functional outcome in progressive ischemic stroke with large vessel occlusion
Source: Front Neurol. 2026 Apr 16;17:1810149. doi: 10.3389/fneur.2026.1810149 (PMC13128660; doi:10.3389/fneur.2026.1810149)
Supplement: Supplementary file 1 [file Data_Sheet_1.pdf]

Supplementary Materials

Supplementary Methods

Derivation of the Stroke Progression Index (SPI) Metric

Design Rationale and Theoretical Basis. The clinical course of acute ischemic stroke is influenced not only by the extent of neurological deterioration, but also by the speed at which deterioration occurs and its severity relative to the baseline neurological status. Based on this concept, we integrated the magnitude of neurological progression, the time required for progression, and relative severity to propose a composite metric termed the Stroke Progression Index (SPI).

Metric Development. We initially started from the most intuitive definition and expressed neurological deterioration as:

$$\mathrm{SPI} = \frac{\triangle \mathrm{NIHSS}}{\Delta \mathrm{t}}$$

However, this linear formulation has two major limitations in clinical application:

Insensitivity to proportional scaling. When ΔNIHSS and Δt increase proportionally across patients (e.g., 4→8 over 2 hours vs. 12→20 over 4 hours), the resulting values remain identical, despite the latter representing a greater cumulative disease burden.

Insensitivity to relative severity. When ΔNIHSS and Δt are identical but baseline NIHSS differs substantially (e.g., 4→8 vs. 16→20 over 4 hours), the linear velocity yields the same value and fails to capture the differing clinical implications of the same absolute change across distinct baseline severities.

Stepwise Mathematical Derivation. Time weighting: sublinear time transformation. To address the limitations of a linear time term under proportional scaling and to more

appropriately reflect the nonlinear influence of time on cumulative risk, we introduced a sublinear time transformation by replacing the denominator Δt with Δt^α (α < 1). In this study, we adopted α = 0.5 as a parsimonious and empirically stable sublinear form:

$$\text{SPI} = \frac{\triangle \text{NIHSS}}{\Delta t^{0.5}}$$

This transformation has two implications. First, it preserves the basic interpretive framework of "change per unit time." Second, by down-weighting the linear contribution of time, it avoids the invariance of the original formulation when ΔNIHSS and Δt increase proportionally, thereby yielding higher values for cases with greater cumulative progression. Notably, the 0.5 exponent is not the only possible choice, but represents a typical sublinear time transformation that is both conceptually reasonable and computationally convenient in practical applications. Alternative sublinear exponents were explored informally and demonstrated similar qualitative behavior, supporting the robustness of this formulation.

Relative burden term: logarithmic severity adjustment. To further differentiate patients with identical ΔNIHSS and Δt but markedly different baseline neurological status, we introduced a relative severity adjustment term:

$$\text{Burden} = \ln\left(1 + \frac{\text{NIHSS}_{\text{progression}}}{\text{NIHSS}_{\text{baseline}}}\right)$$

This logarithmic term quantifies neurological deterioration relative to baseline status, allowing the same absolute NIHSS change to carry different weights across different baseline levels. In addition, the logarithmic transformation compresses extreme values, thereby reducing the influence of outliers and improving the statistical stability of the metric.

Final composite formula. After integrating the time-weighting component and the relative burden adjustment, the final form of SPI was defined as:

$$\text{SPI} = \frac{\triangle \text{NIHSS}}{\Delta t^{0.5}} \ln\left(1 + \frac{\text{NIHSS}_{\text{progression}}}{\text{NIHSS}_{\text{baseline}}}\right)$$

Example calculation. For example, a patient presented with a baseline NIHSS score of 4 at admission and subsequently developed neurological deterioration at 16 hours after symptom onset, with the NIHSS score increasing to 8. Accordingly:

$NIHSS_{baseline} = 4$

$NIHSS_{progression} = 8$

$\Delta NIHSS = 8 - 4 = 4$

$\Delta t = 16$ hours

Substituting these values into the formula yields:

$$SPI = \frac{\triangle NIHSS}{\Delta t^{0.5}} \ln\left(1+\frac{NIHSS_{progression}}{NIHSS_{baseline}}\right) = \frac{4}{16^{0.5}} \ln\left(1+\frac{8}{4}\right) \approx 1.10$$

Summary. Through stepwise derivation, SPI integrates the absolute magnitude of neurological deterioration, the time required for progression, and the severity relative to baseline into a composite metric. By incorporating sublinear time weighting and a logarithmic relative burden adjustment, this metric partially overcomes the limitations of traditional linear velocity formulations and allows a more nuanced characterization of stroke progression over time. From a structural perspective, this modeling approach resembles commonly used expressions for cumulative risk or effect processes in other fields, which may help clarify the rationale underlying its design.

Supplementary Tables

Supplementary Table 1. Univariable Logistic Regression Analysis

Variable	B	SE	Wald	OR	95% CI	P value
Age	0.030	0.012	6.301	1.031	1.007–1.056	0.012
Male sex	-0.536	0.321	2.796	0.585	0.312–1.097	0.095
Hypertension	0.108	0.317	0.117	1.114	0.599–2.073	0.732
Diabetes mellitus	0.536	0.332	2.611	1.709	0.892–3.276	0.106
Hyperlipidemia	-0.465	0.441	1.111	0.628	0.265–1.491	0.292
Atrial fibrillation	0.504	0.499	1.021	1.655	0.623–4.400	0.312
Prior ischemic stroke	0.785	0.350	5.021	2.192	1.103–4.353	0.025
Smoking	-0.461	0.295	2.439	0.631	0.353–1.125	0.118
Anterior circulation occlusion	0.990	0.366	7.338	2.692	1.315–5.512	0.007
Early progression	0.435	0.296	2.162	1.545	0.865–2.761	0.141
EVT	-1.025	0.310	10.916	0.359	0.195–0.659	<0.001
IVT	0.494	0.310	2.534	1.639	0.892–3.013	0.111
$NIHSS_{baseline}$	0.163	0.073	5.005	1.177	1.020–1.357	0.025
$NIHSS_{progression}$	0.173	0.040	19.044	1.189	1.100–1.286	<0.001
ΔNIHSS	0.144	0.040	13.293	1.155	1.069–1.248	<0.001
Δt	-0.007	0.003	4.171	0.993	0.987–1.000	0.041
SPI	0.144	0.040	13.273	1.155	1.069–1.248	<0.001
Length of hospital stay	0.079	0.027	8.421	1.083	1.026–1.142	0.004

Abbreviations: EVT, endovascular treatment; IVT, intravenous thrombolysis; NIHSS, National Institutes of Health Stroke Scale; $NIHSS_{baseline}$, NIHSS score at stroke onset; $NIHSS_{progression}$, NIHSS score at first neurological deterioration; ΔNIHSS, change in NIHSS score from onset to progression; Δt, time from onset to progression; SPI, Stroke Progression Index.

Supplementary Table 2. Collinearity Diagnostics

Variable	Tolerance	VIF	Collinearity assessment
$NIHSS_{baseline}$	0.042	23.821	High collinearity
$NIHSS_{progression}$	0.008	120.558	High collinearity
ΔNIHSS	0.009	112.293	High collinearity
Δt	0.644	1.553	Low collinearity
SPI	0.313	3.194	Low collinearity

Abbreviations: VIF, variance inflation factor; NIHSS, National Institutes of Health Stroke Scale; $NIHSS_{baseline}$, NIHSS score at stroke onset; $NIHSS_{progression}$, NIHSS score at first neurological deterioration; ΔNIHSS, change in NIHSS score from onset to progression; Δt, time from onset to progression; SPI, Stroke Progression Index.

Supplementary Table 3. Multivariable Logistic Regression Analysis Including Anterior Circulation

Variable	B	SE	Wald	OR	95% CI	P value
Age	0.032	0.014	4.999	1.033	1.004–1.063	0.025
Male sex	-0.271	0.385	0.496	0.763	0.359–1.621	0.481
Prior ischemic stroke	0.440	0.411	1.143	1.553	0.693–3.478	0.285
EVT	-1.204	0.378	10.170	0.300	0.143–0.629	0.001
Δt	0.000	0.004	0.007	1.000	0.992–1.009	0.932
Anterior circulation occlusion	1.278	0.448	8.129	3.589	1.491–8.640	0.004
SPI	0.238	0.058	16.985	1.269	1.133–1.421	<0.001

Abbreviations: EVT, endovascular treatment; Δt, time from stroke onset to progression; SPI, Stroke Progression Index.

Supplementary Table 4. Interaction Analysis Between SPI and Anterior Circulation LVO

Variable	OR	95% CI	Wald	P value
SPI	1.214	1.063–1.387	8.209	0.004
Anterior circulation LVO	4.917	1.457–16.601	6.583	0.010
SPI × Anterior circulation LVO	0.960	0.810–1.137	0.226	0.635

Abbreviations: SPI, Stroke Progression Index. LVO, large vessel occlusion.

Supplementary Table 5. Comparison of ROC Models

Model	AUC (95% CI)	ΔAUC vs Age + EVT (95% CI; P)	LRT χ^2 (df), P	Sensitivity %, (95% CI)	Specificity %, (95% CI)
Age + EVT	0.653 (0.574–0.729)	—	—	46.4 (36.2–57.0)	78.3 (69.5–85.1)
Age + EVT + SPI	0.756 (0.684–0.822)	0.103 (0.045–0.180; <0.001)	26.915 (1), <0.001	60.7 (50.0–70.5)	84.0 (75.8–89.7)

Abbreviations: EVT, endovascular treatment; SPI, Stroke Progression Index; LRT, likelihood ratio test.

Supplementary Table 6. Baseline Characteristics of EVT-Treated Patients

Variable	Good outcome (n=78)	Poor outcome (n=42)	P value
Age, years	59.69 ± 12.61	61.86 ± 11.31	0.340
Male sex	62 (79.5)	28 (66.7)	0.185
Hypertension	55 (70.5)	27 (64.3)	0.622
Diabetes mellitus	16 (20.5)	13 (31.0)	0.293
Hyperlipidemia	11 (14.1)	6 (14.3)	1.000
Atrial fibrillation	6 (7.7)	6 (14.3)	0.339
Prior ischemic stroke	9 (11.5)	11 (26.2)	0.072
Smoking	44 (56.4)	19 (45.2)	0.328
Anterior circulation occlusion	50 (64.1)	31 (73.8)	0.380
Early progression	43 (55.1)	29 (69.0)	0.197
IVT	26 (33.3)	11 (26.2)	0.548
Successful reperfusion (mTICI ≥2b)	71 (91.0)	39 (92.9)	1.000
$NIHSS_{baseline}$	3.00 (2.00–4.00)	2.50 (2.00–4.00)	0.953
$NIHSS_{progression}$	10.00 (8.00–13.00)	13.50 (11.25–15.75)	<0.001
ΔNIHSS	7.00 (5.00–10.00)	10.50 (7.25–13.00)	<0.001
Δt, hours	21.00 (5.00–64.75)	11.00 (4.00–43.75)	0.124
SPI	2.76 (1.46–4.13)	4.86 (2.04–9.71)	0.001
Puncture-to-reperfusion time, min	40.50 (33.00–48.75)	39.00 (33.00–45.50)	0.589
ASITN/SIR collateral grade	2.00 (2.00–3.00)	1.00 (1.00–2.00)	<0.001

Values are presented as mean ± SD, median (interquartile range), or n (%). Abbreviations: EVT, endovascular treatment; NIHSS, National Institutes of Health Stroke Scale; ΔNIHSS, change in NIHSS score from onset to progression; Δt, time from onset to progression; SPI, Stroke Progression Index; mTICI, modified Thrombolysis in Cerebral Infarction.

Supplementary Table 7. Univariable Logistic Regression Analysis in EVT-Treated Patients

Variable	B	SE	Wald χ^2	OR	95% CI	P value
Age	0.015	0.016	0.865	1.015	0.984–1.048	0.352
Male sex	-0.661	0.431	2.355	0.516	0.222–1.201	0.125
Hypertension	-0.284	0.407	0.488	0.753	0.339–1.670	0.485
Diabetes mellitus	0.552	0.436	1.605	1.737	0.739–4.082	0.205
Hyperlipidemia	0.015	0.548	0.001	1.015	0.347–2.971	0.978
Atrial fibrillation	0.693	0.612	1.281	2.000	0.602–6.642	0.258
Prior ischemic stroke	1.001	0.499	4.026	2.720	1.023–7.231	0.045
Smoking	-0.449	0.385	1.359	0.638	0.300–1.358	0.244
Anterior circulation occlusion	0.456	0.423	1.164	1.578	0.689–3.615	0.281
Early progression	0.596	0.404	2.180	1.816	0.823–4.008	0.140
IVT	-0.343	0.425	0.650	0.710	0.308–1.633	0.420
Successful reperfusion (mTICI ≥2b)	0.248	0.718	0.119	1.282	0.314–5.238	0.730
$NIHSS_{baseline}$	0.020	0.101	0.039	1.020	0.837–1.244	0.843
$NIHSS_{progression}$	0.120	0.043	7.676	1.127	1.036–1.227	0.006
ΔNIHSS	0.149	0.047	9.961	1.160	1.058–1.272	0.002
Δt	-0.005	0.005	1.328	0.995	0.985–1.004	0.249
SPI	0.165	0.049	11.413	1.180	1.072–1.298	0.001
Puncture-to-reperfusion time, min	-0.004	0.013	0.087	0.996	0.971–1.022	0.768
ASITN/SIR collateral grade	-1.377	0.321	18.389	0.252	0.134–0.473	<0.001

Abbreviations: EVT, endovascular treatment; NIHSS, National Institutes of Health Stroke Scale; ΔNIHSS, change in NIHSS score from onset to progression; Δt, time from onset to progression; SPI, Stroke Progression Index; mTICI, modified Thrombolysis in Cerebral Infarction.

Supplementary Table 8. Collinearity diagnostics (VIF analysis) for the EVT-treated subgroup

Variable	Tolerance	VIF	Collinearity assessment
$NIHSS_{baseline}$	0.581	1.721	Low
$NIHSS_{progression}$	0.174	5.738	Moderate
ΔNIHSS	0.183	5.451	Moderate
Δt	0.587	1.705	Low
SPI	0.354	2.828	Low

Abbreviations: VIF, variance inflation factor; NIHSS, National Institutes of Health Stroke Scale; ΔNIHSS, change in NIHSS score from onset to progression; Δt, time from onset to progression; SPI, Stroke Progression Index;Collinearity was assessed using VIF, with values >5 indicating higher collinearity.

Supplementary Table 9. Sample Sizes Across ASITN/SIR Grades and Progression Timing

ASITN/SIR grade	Early progression, n	Delayed progression, n
1	31	16
2	27	18
3	11	13

Patients with ASITN/SIR grades 0 and 4 were not included because of limited sample size.
